# Medical Opinion and Movement

**Published:** 1906-12-15

**Authors:** 


### Medical Opinion and Movement.

It will be disappointing to general practitioners that Mr. George Brown's motion to make representations to the Privy Council in favour of giving to the registered practitioners resident in England and Wales the power of returning an additional member to the General Council, was again lost. The figures were, however, encouraging—12 voted against the motion and 10 for it, two did not vote, and nine were absent—so that opinion appears now to be almost equally divided on the question.

In the *Outlook* of November 24th there is an interesting account by Dr. Edward Haselt, of Victoria, British Columbia, of the way in which hospitals are managed in that colony. All hospitals receiving sick poor are assisted by Government grants, but the hospitals are open to all, rich or poor. The charges range from seven dollars a week in a public ward to twenty in a private room. If a patient is too poor to pay he has to obtain a card of admission from two directors, and a certificate from a doctor declaring him in need of hospital treatment. The relations of the doctors to the hospital are especially interesting. Any medical man can attend his own patient in a public or private ward, and, according to Dr. Haselt, the hospitals are in no way antagonistic to the interests of the general practitioner, but, on the contrary, are of great assistance and value to him.

In a very masterly report Dr. R. W. Branthwaite, as the inspector under the Inebriates Act for 1905, gives a very complete survey of the working of the reformatories under the Act of 1898, and of the material with which they have to deal. His report shows that whereas the object of the reformatories was chiefly to reclaim habitual drunkards, they have rather become asylums for the segregation of irreformables. This is largely due to the fact that these chronic inebriates belong for the most part to the class of congenital defectives, and their intemperance is an expression of innate feeblemindedness. Dr. Branthwaite condemns the system of the repeated imposition of short terms of imprisonment in this class of individual, and he holds it responsible for considerable mental deterioration. He is of opinion that the only hope for these mental defectives lies in an early committal to the inebriate reformatory, where, by continuous supervision and control, regular habits of living might be inculcated, and the victims might be educated into law-abiding citizens.

Sir Thomas Brooke-Hitching, Mayor of Marylebone, has offered a gift of £1 to the mother of each child born in Marylebone on its attaining the age of one year. The parents must be in receipt of less than £2 per week. Without wishing to depreciate in any way the generous and patriotic impulses which have induced Sir Thomas Brooke-Hitching to follow, in this manner, the lead of Huddersfield, it may be questioned whether such a proceeding is likely to be productive of any permanent good to the community. It may ensure the survival of a certain number of infants, who would otherwise have succumbed to neglect and improper treatment, but that is only a temporary gain. What is required is a means of educating the mothers to a proper sense of their responsibilities, teaching them the first principles of the feeding and care of infants, and making them realise the necessity of a rigid adherence to these principles. On the other hand, we are glad to see that some very good work is being done in the borough of Marylebone, on these lines, by the St. Marylebone General Dispensary, in co-operation with the St. Marylebone Health Society. Courses of lectures are delivered to members in the Hall of Charity, leaflets on the feeding of infants and other matters of health are distributed, and a system of "infant consultations" has been instituted at the Dispensary. We feel sure that more good will be done by these educative influences than by making people dependent upon private charity or municipal benevolence.

The General Medical Council have completed their labours in a four days' session, and during that time they have disposed of a fair amount of work. After a short debate the Council resolved to abolish the possibility of pupilage in the medical profession by erasing the reference to it in their recommendations on education. This has only been done after thorough investigation by the Education Committee, by which the Committee ascertained that little or no use had been made of the provision permitting pupilage for a period of six months in the medical course. Pupilage has not been without its advocates, who claim that the student is enabled thereby to acquire much practical knowledge in dispensing, in the general treatment of patients, and in the business side of general practice. But all these are minor details in comparison with the importance of making the student proficient in all the branches of his profession. Statistics show that 71.5 per cent. of students take six years or more to complete their studies for qualification, and it is only by close contact with all the work and experience offered at a large hospital during that time that the student can acquire the knowledge and skill necessary to equip him for all emergencies. Quite apart from the teaching and laboratory work, the clinical material at the disposal of the student for observation and practice during a few months in a hospital, not only in regard to the number of cases, but also in regard to their variety and severity, is like a concentrated essence of general practice extended over several years. It is on this account that the student is able in a comparatively short space of time to qualify himself for a life-long work. The details of general practice can be readily acquired in the course of an assistantship, taken after the necessary qualifying examinations have been passed. A wider adoption of pupilage would, moreover, tend to foster that system of covering unqualified assistants which is so much to be deprecated. On these accounts, therefore, the Council have shown wisdom in deciding altogether to abolish the period of six months' pupilage as formerly allowed.

